# Reading Specific Small Saccades Predict Individual Phonemic Awareness and Reading Speed

**DOI:** 10.3389/fnins.2021.663242

**Published:** 2021-12-13

**Authors:** Samy Rima, Michael C. Schmid

**Affiliations:** ^1^Department of Sport and Neuroscience, Faculty of Science and Medicine, University of Fribourg, Fribourg, Switzerland; ^2^Bioscience Institute, Faculty of Medical Sciences, Newcastle University, Newcastle upon Tyne, United Kingdom

**Keywords:** vision, microsaccades, eye-tracking, phonemic awareness, reading speed

## Abstract

Small fixational eye-movements are a fundamental aspect of vision and thought to reflect fine shifts in covert attention during active viewing. While the perceptual benefits of these small eye movements have been demonstrated during a wide range of experimental tasks including during free viewing, their function during reading remains surprisingly unclear. Previous research demonstrated that readers with increased microsaccade rates displayed longer reading speeds. To what extent increased fixational eye movements are, however, specific to reading and might be indicative of reading skill deficits remains, however, unknown. To address this topic, we compared the eye movement scan paths of 13 neurotypical individuals and 13 subjects diagnosed with developmental dyslexia during short story reading and free viewing of natural scenes. We found that during reading only, dyslexics tended to display small eye movements more frequently compared to neurotypicals, though this effect was not significant at the population level, as it could also occur in slow readers not diagnosed as dyslexics. In line with previous research, neurotypical readers had twice as many regressive compared to progressive microsaccades, which did not occur during free viewing. In contrast, dyslexics showed similar amounts of regressive and progressive small fixational eye movements during both reading and free viewing. We also found that participants with smaller fixational saccades from both neurotypical and dyslexic samples displayed reduced reading speeds and lower scores during independent tests of reading skill. Slower readers also displayed greater variability in the landing points and temporal occurrence of their fixational saccades. Both the rate and spatio-temporal variability of fixational saccades were associated with lower phonemic awareness scores. As none of the observed differences between dyslexics and neurotypical readers occurred during control experiments with free viewing, the reported effects appear to be directly related to reading. In summary, our results highlight the predictive value of small saccades for reading skill, but not necessarily for developmental dyslexia.

## Introduction

Small fixational eye-movements, such as microsaccades, quickly reposition the image within the fovea. They have been shown to be a direct manifestation of the deployment of covert attention (Engbert and Kliegl, 2003; Pastukhov and Braun, 2010; Yuval-Greenberg et al., 2014; Lowet et al., 2018). The role of these eye movements during natural vision remains debated. While visual discrimination is enhanced at the target location of a microsaccade for example, it is impaired at the diametrically opposite location (Shelchkova and Poletti, 2020). Such miniature eye-movements also seem to be necessary to compensate for inhomogeneities of spatial resolution within the foveola (Poletti et al., 2013). Beyond the fovea, they have been shown to contribute to high acuity vision (Martinez-Conde et al., 2000, 2009). From these observations it is therefore natural to assume that microsaccades could be beneficial for visually demanding tasks such as reading of printed text, though our understanding of this relationship is still very limited.

There is very little knowledge about the pattern of microscopic eye-movements of reading impaired individuals during reading. Studies of eye movements during reading in dyslexics for example, report smaller than average saccade amplitudes, longer fixation times and more regressive saccades (Eden et al., 1994; Biscaldi et al., 1998; Prado et al., 2007; Mozzachiodi and Byrne, 2010; Trauzettel-Klosinski et al., 2010; Hatzidaki et al., 2011; Bellocchi et al., 2013). The abnormal characteristics of eye-movements during reading in dyslexics have been linked with spatiotemporally impaired processing of foveated words (Rayner, 1998; Hari et al., 1999; Conlon et al., 2004; Bosse et al., 2007; Lallier et al., 2010; Vidyasagar and Pammer, 2010; Stenneken et al., 2011; Lobier et al., 2012; Bellocchi et al., 2013; Tiadi et al., 2016). Such oculomotor instability also manifests in dyslexics as poorly coordinated vergence (Kirkby et al., 2008) and higher and more variable fixation disparity (Jainta and Kapoula, 2011) during real text reading, potentially leading to perceived letter overlap. Poor binocular coordination was found in dyslexic children while reading single words (Bucci et al., 2008). Later, the same group described higher disconjugacy during smooth pursuit in dyslexics but only in the rightward direction (Yang et al., 2010), possibly linked to the reading deficit which follows the same directionality. Microsaccade frequencies have been found to be similar in dyslexics and neurotypical readers during non-verbal perceptual tasks (Wilcockson et al., 2019; Rima et al., 2020), however, their properties and their relationship with reading abilities, have never been investigated during natural reading. Quantifying the role of fixational eye-movements and their relationship with poor reading is important to understand multifactorial reading disorders like dyslexia. To isolate causes of such fixational instability, it is also necessary to compare fixational eye movements during reading with other tasks, such as free viewing of scenes.

In this study, we investigated therefore the properties of small fixational saccades during reading, in age and education matched neurotypical and dyslexic populations. Our main, previously undescribed result is that the increased occurrences of small saccades during reading but not free viewing, and their increased spatio-temporal variability predict low reading speeds and phonological awareness. While neurotypicals and dyslexics did not show any significant difference in the occurrences or the spatial and temporal entropies of small, in either reading or free viewing, slow readers from both populations had a higher probability of having more small saccades with larger spatial and temporal entropies during reading. reading speeds and phonological awareness scores. The absence of these differences in good and slow readers during free viewing, likely indicates that deficient oculomotor planning in dyslexics is specific to reading.

## Materials and Methods

### Subjects

Twenty-eight monolingual native English speakers were initially recruited for this study. Of them, 14 participants were previously diagnosed with developmental dyslexia (9 females and 6 males, age: *M* = 21.64, SD = 5.37). The remaining (neurotypical group) 14 participants had no underlying disorders (7 females and 7 males, age: *M* = 22.35, SD = 5.86). We recruited the participants through an advertisement posted on a Newcastle University social media page. Each participant was paid £30 in Amazon vouchers to compensate for their time. Participants gave written informed consent before the experiment began and confirmed that they were not taking any medication that could affect reaction times, were not a recovering alcoholic, and had no history or neurological or psychiatric disease. The local ethics committee at Newcastle University approved the ethics for the current research.

### Apparatus

We acquired all oculomotor measurements in a dimly illuminated, quiet room. The experiment was controlled with the EventIDE software^1^. Stimuli were displayed on a on a gamma corrected VIEWPixx monitor with 1920 × 1080 resolution at a refresh rate of 120 Hz and a diagonal length of 60.5 cm. Subjects sat at a fixed distance of 62 cm from the monitor, and their heads were stabilized with a chin rest. Eye movements were recorded from both eyes at 500 Hz/eye using an EyeLink 1,000 eye-tracker (instrument noise 0.01 deg RMS).

### Task and Procedure

#### The Dyslexia Adult Screening Test

**FIGURE 1:**
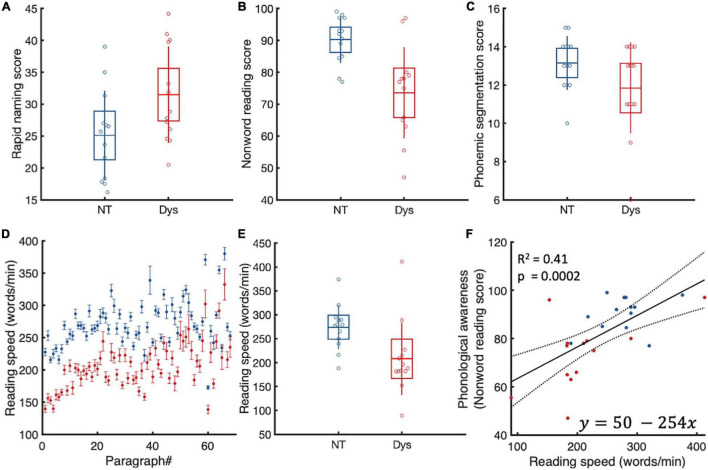
Dyslexia adult screening test scores. **(A)** Rapid naming. Dyslexics (red) are slower and make more mistakes during rapid naming. **(B)** Non-word reading. Dyslexics (red) are slower at reading the passage and have more difficulty pronouncing reading non words. **(C)** Phonemic segmentation. Dyslexics were slower and had more difficulty manipulating phonemes. **(D)** Reading speed. As expected, dyslexics were slower at reading the same material as neurotypicals. **(E)** Only non-word reading predicted reading speeds. Dyslexics are in red. Boxes represent standard deviations. **(F)** Linear regression analysis of reading speed and phonological awareness. As expected faster readers had better phonological skills.

Each participant completed three subtests of the Dyslexia Adult Screening Test (Harrison and Nichols, 2005). The subtests that were carried out in the current study were Nonsense Passage Reading, Phonemic Segmentation, and Rapid Naming. All comparisons yielded significant differences between neurotypical readers and dyslexics (Figure 1). Linear regression analysis showed that only non-word reading scores predicts reading speed. Readers with better phonological awareness were also likely to be faster readers.

#### Short Story Reading

In this task, participants had to read a short story. The short story was divided into 68 paragraphs. The length of paragraphs ranged from 4 to 11 lines (*M* = 6.11, SD = 1.26) and 46 to 108 words (*M* = 64.05, SD = 12.59). The font was Adobe Caslon, and the font size was 16p. Each letter was around 0.5 degrees of visual angle (Legge and Bigelow, 2011). Participants had as much time to read the paragraph as they wanted, and they pressed space to read the subsequent paragraph.

#### Reading Speed

We estimated individual reading speeds for each paragraph by dividing the number of words in a paragraph by the time it took the subject to read the paragraph. As expected, our dyslexic population had lower reading speeds compared to neurotypicals in 68/68 paragraphs. On the group level (Figure 1D), dyslexics had 1.4x slower reading speeds on average (NT: Mean = 275 WPM (± 46.2 SD, Dys: Mean = 209 WPM (± 75.91 SD), Wilcoxon’s *p* = 0.003).

#### Free Viewing of Natural Scenes

Stimuli consisted of 71 natural scenes, for which the RGB values for luminance were equalized. All images were centered on the screen and subtended 26 × 18 degrees of visual angle. For each participant the experiment started with a 5-point GLM calibration procedure, which calculates two pairs of linear coefficients for mapping the tracker input into the screen coordinates. The start of each free viewing trial required the participant to fixate on a central red cross, overlaid on a black background for 500 ms. After this period, the cross would become green, and the participant would have to maintain an additional 700 ms of fixation before the scene would appear. The participant then had 10 sec to explore freely each scene once.

#### Eye Movement Detection

We detected binocular small saccades using a modified version of the algorithm provided in Otero-Millan et al. (2014). We performed multiple steps to clean the data of blinks and non-saccadic eye movements from the reading and free viewing datasets.

**FIGURE 2:**
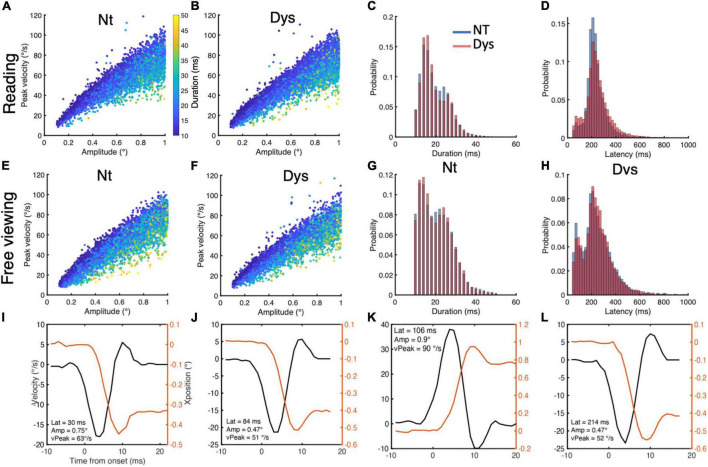
Small saccade characteristics during reading and free viewing. Small saccade reading main sequence for neurotypicals **(A)** and dyslexics **(B)**, with duration overlay. **(C)** Small saccade duration distributions during reading for neurotypicals (blue) and dyslexics (red). **(D)** Small saccade latency distributions during reading for neurotypicals (blue) and dyslexics (red). Small saccade free viewing main sequence for neurotypicals **(E)** and dyslexics **(F)**, with duration overlay. **(G)** Small saccade duration distributions during free viewing for neurotypicals (blue) and dyslexics (red). **(H)** Small saccade latency distributions during free viewing for neurotypicals (blue) and dyslexics (red). **(I–L)** Positions and peak velocity profiles for small saccades of different latencies, amplitudes, and peak velocities.

– Eye movements that had a $\frac{acceleration_{peak}}{deceleration_{peak}}=1$ were identified as blinks and removed.
– Eye movements with *deceleration*_*peak*_ and *acceleration*_*peak*_>40000°/s^2^ were removed.
– Eye movements with durations <10 ms and >100 ms were removed.
– Eye movements with amplitudes <0.1 were removed.
– Eye movements with latencies < 50 ms & > 1000 ms were removed (Figure 2D for reading and Figure 2H for free viewing).

The main sequence for small saccades during reading is shown in Figure 2A for neurotypicals and Figure 2B for dyslexics, with duration as a color overlay. The main sequence for small saccades during free viewing is shown in Figure 2E for neurotypicals and Figure 2F for dyslexics, with duration as a color overlay. Saccades below the 100 ms latency mark had stereotypical saccade position and velocity profiles. The position and velocity profiles of small saccades with different amplitude, peak velocity and latencies are shown in Figures 2I–L. For the reading task, we included 7,391 small saccades from the neurotypical population and 11,726 small saccades from the dyslexic population. For the free viewing task, we included 6,213 small saccades from the neurotypical population and 5556 small saccades from the dyslexic population.

#### Estimating Landing Position Variability of Small Fixational Saccades

To estimate the spatial variability of fixational saccade landing positions, we employed the concept of entropy (Shannon, 1948). To calculate spatial entropy, we plotted the landing positions of small saccades for each subject as a bivariate histogram of x and y positions in visual degrees, giving an image of saccade landing point spatial distribution with intensity representing landing point probability. We then calculated the entropy of each image using MATLAB’s entropy function, which applies the following equation:


$$\text{Entropy}=-\sum \left(p\times \log_{2}p\right)$$

Where, p represents the histogram counts of the bivariate histogram representation of landing positions.

#### Estimating Temporal Variability of Small Fixational Eye-Movements

The temporal variability of small fixational eye movements can be considered as the compound variability of latencies and temporal variability of occurrence with respect to start and end of a paragraph. To estimate the temporal variability of small saccades, we plotted individual saccade latencies as a function of saccade normalized temporal occurrence within a paragraph, for each subject, as a bivariate histogram of x and y positions. The counts of this bivariate histogram were the used to calculate entropy with the same equation used for spatial entropy.

#### Linear Regression Analysis

All linear regression analyses were performed on the level of subjects after averaging through paragraphs. To reduce the effects of outliers we used robust fitting with bisquare weight function with a tuning constant of 4.685. We also estimated the contribution of each subject to the linear regression by comparing fit parameters before and after excluding them from the analysis.

## Results

### Small Saccade Ratios, Spatial and Temporal Entropy, Predict Reading Speeds

**FIGURE 3:**
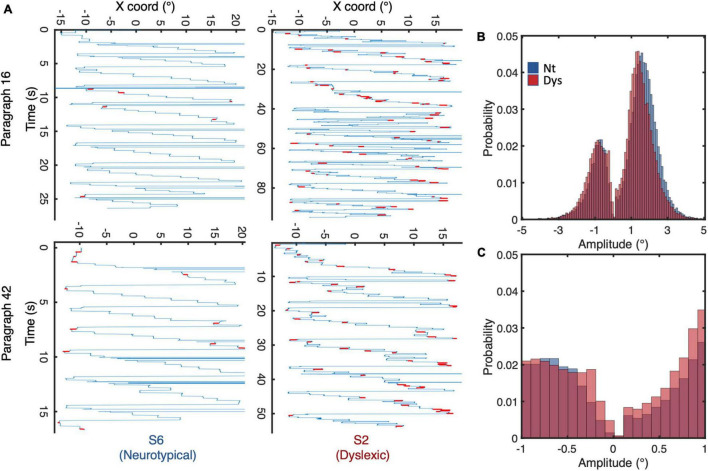
**(A)** Eye movement scan paths of a neurotypical reader (left panel) and dyslexic reader (right panel) while reading identical paragraphs of a short story. **(B)** Saccadic amplitude distributions for neurotypicals (blue) and dyslexics (red) during reading. **(C)** Distribution of saccade amplitudes <1. Negative/positive amplitudes indicate saccades to the left/right of fixation, respectively.

From the 28 recruited subjects, only 13 neurotypicals and 13 dyslexics completed all tasks and whose results will be subsequently interpreted and discussed. Viewing the raw eye traces of a neurotypical individual reading text (Figure 3A left panels) shows the typical regular text scanning saccade pattern: progressive saccades occur very regularly in the reading direction with large regressive saccades between line transitions. Saccades <1 (highlighted in red) occur occasionally during fixation periods. This pattern can be profoundly different in certain dyslexics (right panels of Figure 3A), the overall appearance of the scan pattern has a noisier appearance, with shorter and more variable amplitudes and less predictable spatial and temporal occurrences, resulting in a higher density of fixation periods and occurrences. As expected, this eye movement pattern was associated with an extended reading duration for the dyslexic individuals. The following analyses are aimed at quantifying and summarizing these effects for different small saccade parameters for neurotypical vs. dyslexic study participants.

Overlaying the amplitude distributions of all saccades of neurotypical readers and dyslexics, we observe a general shift of the curve toward shorter amplitudes (Figure 3B). The median amplitude of progressive saccades in neurotypical was 2° with a variance of 1.1°, while that of dyslexics was 1.7° with a variance of 1.4°. Zooming in on the amplitude probability distribution of saccades <1 (Figure 3C), reveals different probability distribution profiles between neurotypical readers and dyslexics on one hand, and progressive (positive amplitude) vs regressive (negative amplitude) saccades on the other.

**FIGURE 4:**
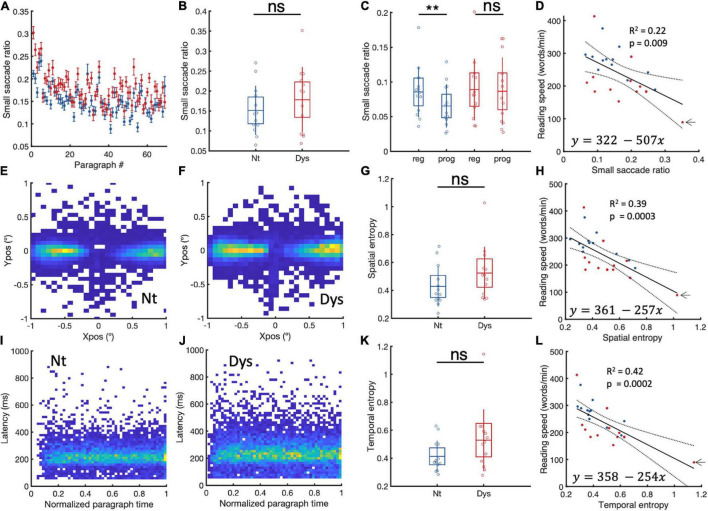
Small saccade occurrences and spatiotemporal entropy. **(A)** Paragraph specific small saccade ratios in neurotypicals (blue) and dyslexics (red). **(B)** Subject specific small saccade ratios for neurotypicals (blue) and dyslexics (red). **(C)** Comparison between regressive and progressive subject specific small saccade ratios for neurotypicals (blue) and dyslexics (red). **(D)** Linear regression analysis of small saccade ratios and reading speed. Small saccade landing point distributions for **(E)** neurotypicals and **(F)** dyslexics. **(G)** Subject specific small saccade spatial entropy for neurotypicals (blue) and dyslexics (red). **(H)** Linear regression analysis of small saccade spatial entropy and reading speed. **(I,J)** Small saccade temporal occurrences in neurotypicals and dyslexics, respectively. **(K)** Subject specific temporal entropy for neurotypicals (blue) and dyslexics (red). **(L)** Linear regression analysis of temporal entropy and reading speed. *p < 0.05, **p < 0.01, and ***p < 0.001. ns > 0.05 (non-significant).

Small saccade proportions were calculated as the ratio of saccades <1 to all occurring saccades. Dyslexics show higher small saccade proportions in 55/68 paragraphs (Figure 4A). On the group level (Figure 4B.), irrespective of saccade direction (0.1–1°), we found no significant difference in small saccade occurrence between dyslexics and neurotypicals, (NT: Mean = 0.15 (± 0.06 SD), Dys: Mean = 0.18 (± 0.08 SD), Wilcoxon’s *p* = 0.48). In their study (Bowers and Poletti, 2017), show that readers had more regressive compared to progressive small saccades. We find evidence for this bias toward regressive saccades in neurotypicals (Figure 4C, Reg = 0.085% (±0.03SD), Prog = 0.065% (±0.031SD), Wilcoxon’s sign rank *p* = 0.02). Surprisingly, this was not the case in dyslexics who showed on average similar quantities of regressive vs progressive saccades (Figure 4C, Reg = 0.089% (±0.044SD), Prog = 0.086% (±0.024SD), Wilcoxon’s sign rank *p* = 0.2), corroborating previous results of increased numbers of progressive saccades in dyslexics while reading (Prado et al., 2007; Nárai et al., 2021). These results suggest that the main difference between neurotypicals and dyslexics in the occurrences of small saccades is due to the occurrence of more progressive fixational eye movements during reading in dyslexics. We then performed a linear regression analysis to verify the relationship between reading speed and small saccade occurrences (Figure 4D). The analysis reveals that small saccade ratios predict reading speeds. Subjects with higher small saccade ratios have higher probabilities of being slow readers, irrespective of whether they have been classified as neurotypical or dyslexic. To control for the contribution of subjects with extreme values on the goodness of fit, we reran the analysis iteratively by leaving one participant out at each iteration; removing the subject indicated by the arrow decreased the goodness of fit (*R*^2^ = 0.09, *p* = 0.07). The Pearson correlation, however, still showed a significant negative relationship between small saccade ratio and reading speed even after the removal of that subject (Pearson’s *r* = −0.4, *p* = 0.035, 95%CI = [−0.69, −0.026]). These results indicate that while it is not possible to differentiate neurotypicals from dyslexics in terms of small saccade occurrences, these occurrences still contribute to individual reading speeds.

We wondered whether spatiotemporal distribution of small saccades landing points might be a contributing factor to slow reading. To capture spatial variability in both its horizontal and vertical dimensions, we measured the variability of spatial distribution of landing position by estimating subject specific landing position entropy (see section “Materials and Methods”). The larger the entropy, the more variable the landing positions. Group-specific cumulative landing point distributions for neurotypicals and dyslexics subject are plotted in Figure 4E,F. In Figure 4G we can see that dyslexics tend to have higher spatial entropies compared to neurotypicals, however, this difference was not statistically significant (NT = 0.42 ± 0.14, Dys = 0.52 ± 0.18, Wilcoxon’s *p* = 0.13). A robust linear regression (Figure 4H) with individual landing point entropy as predictor and reading speed as response shows that subjects with higher spatial entropies are more likely to be slow readers (tstat = −4, *R*^2^ = 0.39, *p* = 0.0003). To control for the contribution of subjects with extreme values on the goodness of fit, we reran the analysis iteratively by leaving one participant out at each iteration; removing the subject indicated by the arrow decreased the goodness of fit (*R*^2^ = 0.27, *p* = 0.004). The Pearson correlation still showed a significant negative relationship between spatial entropy and reading speed even after the removal of that subject (Pearson’s *r* = −0.63, *p* = 0.0005, 95%CI = [−0.82, −0.32]).

We then investigated the temporal dimension of small saccades. First looking at small saccade latencies, we hypothesized that increased small saccade occurrences could hint at longer times between two successive regular saccades. However, the latencies were not significantly longer for dyslexics compared to neurotypicals (NT: Mean = 225 ms (± 12 SD), Dys: Mean = 232 ms (± 15 SD), Wilcoxon’s *p* = 0.24). We also did not find a significant relationship between intersaccadic intervals and small saccade occurrences (coefficient estimate = −0.0003, tstat = 0.2, *R*^2^ = −0.03, *p* = 0.75). We then performed a temporal entropy analysis like the one performed for the spatial distribution of landing points (see section “Materials and Methods”).

Group-specific cumulative temporal distributions (latency vs temporal occurrence relative to start of paragraph) for neurotypicals and dyslexics subject are plotted in Figure 4I for neurotypicals and Figure 4J for dyslexics. In Figure 4K we can see that while dyslexics tended to have higher temporal entropy compared to neurotypicals, this difference was overall not statistically significant (NT = 0.41 ± 0.11, Dys = 0.52 ± 0.22, Wilcoxon’s *p* = 0.14). A robust linear regression (Figure 4L) with individual temporal entropy as predictor and reading speed as response showed that subjects with higher small saccade temporal entropy were more likely to be slow readers (tstat = −4, *R*^2^ = 0.42, *p* = 0.0002). To control for the contribution of subjects with extreme values on the goodness of fit, we reran the analysis iteratively by leaving one participant out at each iteration; removing the subject indicated by the arrow decreased the goodness of fit (*R*^2^ = 0.23, *p* = 0.009). The Pearson correlation still showed a significant negative relationship between spatial entropy and reading speed even after the removal of that subject (Pearson’s *r* = −0.63, *p* = 0.0006, 95%CI = [−0.82, −0.32]).

### Free Viewing

**FIGURE 5:**
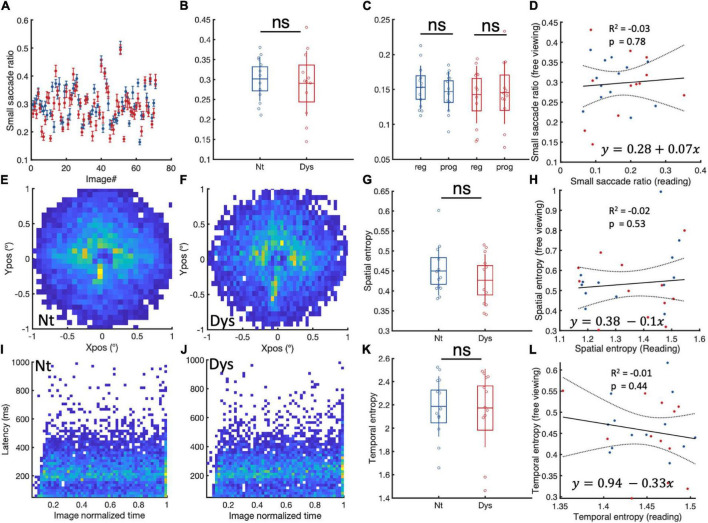
Small saccade occurrences and spatiotemporal entropy in free viewing. **(A)** Paragraph specific small saccade ratios in neurotypicals (blue) and dyslexics (red). **(B)** Subject specific small saccade ratios for neurotypicals (blue) and dyslexics (red). **(C)** Comparison between regressive and progressive subject specific small saccade ratios for neurotypicals (blue) and dyslexics (red). **(D)** Linear regression analysis of small saccade ratio and during reading and free viewing. **(E,F)** Small saccade landing point distributions for neurotypicals and dyslexics, respectively. **(G)** Subject specific small saccade spatial entropy for neurotypicals (blue) and dyslexics (red). **(H)** Linear regression analysis of small saccade spatial entropy during reading and free viewing. **(I,J)** Small saccade temporal occurrences in neurotypicals and dyslexics, respectively. **(K)** Subject specific temporal entropy for neurotypicals (blue) and dyslexics (red). **(L)** Linear regression analysis of small saccade temporal entropy and during reading and free viewing. ns > 0.05 (non-significant).

With such marked small fixational saccade patterns during reading, we wondered to what extent these eye movement patterns might also be observable outside the context of visual text processing. To this end, we analyzed free viewing data from the same 13 neurotypicals and 12 dyslexics, as the data file from one dyslexic subject was corrupted. During the task which required free viewing of natural scenes, neurotypicals showed a higher ratio of small saccades (<1) compared to dyslexics in 47/71 images (Figure 5A). On the group level Figure 5B, however, there was no significant difference between the two populations (NT: Mean = 0.3 (± 0.05 SD), Dys: Mean = 0.29 (± 0.08 SD), Wilcoxon’s *p* = 0.76). Neurotypicals and dyslexics showed similar ratios of leftward vs rightward small saccades (Figure 5C). Furthermore, there was no relationship (Figure 5D) individual occurrences of small saccades during reading and free viewing of scenes (coefficient estimate = 0.07, tstat = 0.2, *R*^2^ = −0.03, *p* = 0.78).

The spatial entropies of small saccades during free viewing (Figures 5E–G) were also not different between neurotypicals and dyslexics. A linear regression analysis of individual small saccade entropies during free viewing and reading showed no relationship between these two measures (Figure 5H). The same observation was made for small saccade temporal entropies during free viewing (Figures 5I–L).

### Phonological Skill and Spatio-Temporal Characteristics of Small Saccades

Previous studies showed that reading comprehension depends heavily on phonemic awareness (Engen and Høien, 2002). We measured phonological awareness using a non-reading test where subjects had to read a passage containing words created from frequent phonemes of the English language but did not exist in the dictionary. Good performers on this test show the ability to manipulate individual phonemes to read new words that they hadn’t previously encountered. Our results on this test differentiate well between neurotypicals and dyslexics and slow and fast readers. We performed linear regression analyses to verify the relationship between individual phonological awareness scores and small fixational eye movements during reading and free viewing. We found that small saccade ratio, spatial and temporal entropies were reliable predictors of phonological awareness.

**FIGURE 6:**
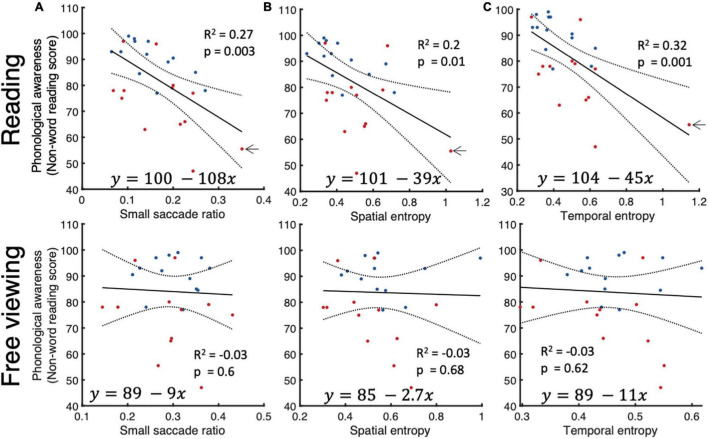
Relationship between phonological awareness and small saccade parameters. Linear regression analysis between phonological awareness scores on one hand and small saccade ratios **(A)**, **(B)** spatial entropy, and **(C)** temporal entropy on the other for reading (top row) and free viewing (bottom row).

The presence of subjects with very low scores of phonological awareness, or high spatial and temporal entropies, however, could either be outliers due to measurement error, or cases of severe dyslexia. The subject indicated by an arrow in Figures 6A–C, could be such a subject. After excluding this subject from the analysis, we found that the significant relationship between phonological awareness on hand and spatial and temporal entropies on the other remained but was canceled for small saccade ratio. We did not detect any significant relationship between small saccade parameters in free viewing and scores of phonological awareness.

## Discussion

### Result Summary

In this study we investigated the relationship between small fixational saccades (<1) and reading skill. To that end, we recorded binocular eye-movements during a natural reading task. Data was acquired from 26 subjects: 13 neurotypical adult readers and 13 reading impaired adults, previously diagnosed as dyslexics. Our results revealed that neurotypicals and dyslexics had no significant differences in either the occurrences, the spatial or the temporal variability of small saccades. The only aspect that made small saccades in dyslexics clearly distinguishable from those in normal readers was a higher proportion of progressive saccades. At the same time saccade frequency and spatio-temporal variability predicted reading speed and phonological awareness, two key aspects of reading that are impaired in dyslexia. Readers that had higher occurrences of small saccades and whose small saccades were less spatially and temporally predictable, were more likely to be slow readers with poor phonological awareness. These effects appear to be specific to reading, as both neurotypical and dyslexic subjects showed no differences in either occurrence, spatial or temporal distributions of small saccades during free viewing of scenes. No correlation was found between these measures and individual phonological skills. In what follows we first discuss these findings considering the recent literature and follow with considerations on the consequences of small fixational eye-movements for reading.

### Comparison With Previous Studies

Inadequate oculomotor control has been associated with dyslexia for quite some time (Pavlidis, 1981). There has been, however, no consensus, as some research groups reported oculomotor differences between dyslexics and controls (Stein et al., 1988; Biscaldi et al., 1994, 1998; Eden et al., 1994), while others didn’t (Olson et al., 1983; Stanley et al., 1983; Ygge et al., 1993). Within the rich field of oculomotor research, comprehensive accounts of small fixational saccades during reading are rare. A recent study investigated microsaccades (saccades <0.5) during visual scanning of text (Bowers and Poletti, 2017). These authors reported that high microsaccade rates predicted slow reading in subjects considered to be regular readers. Our study replicates this important finding and extends it to individuals with developmental dyslexia. Characterizing the spatial layout of microsaccades, Bowers and Poletti (2017) also reported more backward microsaccades as opposed to forward saccades during reading. A similar finding was also obtained by Kowler and Anton (1987). Our results in typical readers confirm this bias toward more backward small saccades, including microsaccades. Importantly, however, our findings also show that individuals with reading deficits do not present this bias toward regressive small saccades.

Bowers and Poletti suggested that task-relevant microsaccades are the ones that start and end on the line of text while task-irrelevant don’t. Although our data don’t allow us to determine the landing points of saccades relative to single characters, the spatial variability of small saccades calculated using Shannon entropy shows that such reading relevant “microsaccades” might not be the norm. Here, a higher spatiotemporal entropy of microsaccades in impaired readers is linked to lower reading speeds and poor phonological skills. To reconcile our findings with the ones from Bowers and Poletti on microsaccade patterns, we speculate that reading-relevant microsaccades might benefit reading, whereas increased microsaccade spatio-temporal variability might harm it. Such detrimental effect of the increased spatial variability in the landing points during reading has also been suggested in a recent study (Franzen et al., 2021).

While we cannot entirely determine the perceptual consequences of microsaccades at this point, we follow with a discussion on their potential benefits vs harms for reading.

### Microsaccades as a Mechanism to Increase the Perceptual Span During Reading?

During reading, the spatial area from which we extract helpful information during a single fixation is limited. It consists of 3–4 letters to the left of fixation and 14–15 letters to the right of fixation in skilled readers of languages read from left to right. The spread and asymmetry of this effective reading range, or perceptual span (Rayner et al., 2010), reflect individual language ability (Choi et al., 2015) as well as reading and spelling skills (Veldre and Andrews, 2014). Reading speed (words/minute) and perceptual span are intertwined (Rayner et al., 2010; Ashby et al., 2012) fast readers present a larger perceptual span than slow readers.

Bowers and Poletti (2017) hypothesized that microsaccades in slow readers had the purpose of increasing the available perceptual span for identifying words that fall outside the fovea. The presumption here is that microsaccades improve perception (Pastukhov et al., 2013; Poletti et al., 2013; Yuval-Greenberg et al., 2014; Lowet et al., 2018; Shelchkova and Poletti, 2020). Accordingly, microsaccades would constitute a compensation mechanism for poor readers to optimize their access to rightward parafoveal words by making more progressive saccades. Consequently, poor readers would have an increased reading time from the need to perform more eye movements per unit of text.

We hypothesize that what allows readers to be quick is their ability to deploy a wide and temporally stable perceptual span. Regular and efficient parallel sampling properties allow for the extraction of more than one word at a time. Reading a sentence is then done with fewer fixations and larger saccades. We argue that a small and temporally irregular perceptual span might emerge from small fixational eye movements with high spatiotemporal entropy. A perceptual span inefficient at processing the currently fixated word and its immediate neighbors would slow down contextualization in the sentence or even a text. While it might be true that slower readers employ progressive, small fixational saccade to temporarily increase their perceptual span, our data indicates the likely existent of temporally and spatially erratic small fixation saccades that are irrelevant to reading. As such, an inefficient perceptual span might lead to more frequent text-relevant progressive saccades to recapture previously fixated but poorly integrated information, but also to more frequent text-irrelevant small saccades.

Both in neurotypicals and dyslexics, slower readers showed an increased number of small fixational saccades and reduced phonemic awareness scores. Might there be a common dysfunctional mechanism that underlies both poor phonemic awareness and inefficient visual perception? In a longitudinal study, Lyytinen et al. (2015) followed Finnish children from birth to puberty. They demonstrate that children of parents with poor phonological abilities will display comparable difficulty in discriminating similar but different sounds, even before learning how to read. These children’s reading skills were correlated with event-related potentials in response to vowel duration changes within consonant-vowel syllable sounds. These results suggest a hereditary genetic component of poor phonological awareness. Comparatively, no study to the best of our knowledge has investigated whether infants’ initial visual information extracting skills and fixational stability are also hereditary and whether they predict future reading skill.

### Is the Smaller and Instable Perceptual Span of Poor Readers a Consequence of Saccadic Suppression of Word Encoding?

The eye can only process fine print through the foveal and close parafoveal region of the eye. Disruption of fixational stability through small, unplanned eye movements might hinder the proper extraction of detailed visual evidence by randomly shifting and resetting the perceptual span’s locus.

The role of microsaccades in improving visual perception seems to be task dependent. Loughnane et al. (2018) showed that microsaccade generation impedes visual encoding, which delays the accumulation of visual evidence that enables quick decision making. Furthermore, microsaccade occurrence seems to be associated with a compression of both the spatial and temporal dimensions of visual perception (Hafed, 2013; Yu et al., 2017). During fixation, the onset of a visual stimulus leads to a 200 ms decrease of microsaccade occurrence, likely to allow for correct inspection and uninterrupted processing of new visual information (Rolfs, 2009).

The suggestion that unplanned, spatially, and temporally more erratic eye movements stems from the finding that microsaccade onset suppresses visual activity in the superior colliculus (Hafed and Krauzlis, 2010; Rolfs and Ohl, 2011). Similarly, a decrease of activity in V1 and an increase of activity in V4 of macaque monkeys accompany the occurrence of a microsaccade during fixation (Leopold and Logothetis, 1998; Hass and Horwitz, 2011). This suppression in early visual centers and the increase in activity in attention-related area V4, could indicate a re-mapping of the cortical retinotopic representation of the visual stimuli after their displacement due to eye movements. If an unplanned microsaccade occurs during reading, then re-mapping the visual representation of words could hinder the word’s ongoing processing and might even restart it. As a result, slow readers or individuals with developmental dyslexia who display more microsaccades than neurotypical individuals might experience heightened difficulty with word encoding.

The perceptual span’s size increases with reading skill and experience, allowing readers to make fewer fixations to read a text, subsequently allowing faster reading. The emergence of the perceptual span can be thought of as the result of a process of increasing the prediction of which words might come after the currently fixated word. The spatio-temporal regularity of the perceptual span would be then linked to the how well readers can preview words in their parafovea (Veldre and Andrews, 2018) and decrease the necessity to fixate them. This would manifest in larger saccades and increased reading speed and stability during fixation of words in normally developing readers. In the case of reading acquisition difficulties, inefficient word encoding due to fixational instability would subsequently hinder the parafoveal preview (Silva et al., 2016) of upcoming words, making it harder for people with dyslexia to plan the next eye movement. The development of the parafoveal preview benefit might be linked to how well a person can learn the inherent statistics of the information baring visual stimulus (Veldre and Andrews, 2018). Increasing evidence for statistical learning impairment in dyslexia (Kahta and Schiff, 2016; Dobó et al., 2021), and notably in the visual domain (Sigurdardottir et al., 2017; Singh et al., 2018), stresses the potential of visual perceptual learning in improving reading skills of dyslexic individuals (Gori and Facoetti, 2014; Meng et al., 2014; Wang et al., 2014).

What could be the neural origin of microsaccades interfering with reading? A recent report demonstrated that activity in the left fusiform gyrus, left precentral and left superior temporal sulcus predicted reading ability in both French and Chinese readers (Feng et al., 2020). Reading specific regions near the occipital (fusiform gyrus) and inferior parietal lobe overlap visual maps, most likely for attentional guidance during reading (Sood and Sereno, 2016). Furthermore it has recently been shown that visual word form area (Dehaene and Cohen, 2011) located in the fusiform gyrus is, in addition to being part of the language circuitry, also an inherent part of the attention circuitry (Vogel et al., 2012; Chen et al., 2019). Poor communication between visual word identity coding areas (fusiform) and attention guiding areas (parietal) could manifest in attentional deficits and poor oculomotor control. Other studies which have investigated the fixational stability and pursuit capabilities of dyslexic children, suggest an immaturity of brain structures involved directly in oculomotor control such as the superior colliculus, the cerebellum, and the frontal eye fields. Thus, even before thinking about reading abilities, it would be pertinent to consider actively developing the capabilities of proper oculomotor control such as fixational stability and saccade precision. A recent review by Bucci (2019) provides impactful arguments about the potential of computer-based visual training to improve the attentional and subsequently the oculomotor capabilities of dyslexic children. Additionally, recent studies (Antzaka et al., 2017; Franceschini et al., 2013, 2017) indicate that using first-person shooter games that require strategic deployment of visual attention and capabilities of inhibitory control to identify relevant targets and ignore distractors could improve dyslexic subjects reading skills.

### Limitations

A limitation of our study is the use of a video-based eye-tracker with relatively low spatial resolution (Kimmel et al., 2012). Because of this, artifacts in eye position signal due to small head movements and pupil size change are more likely (Nyström et al., 2016). These factors limit our analysis of microsaccade direction decreasing our confidence in which microsaccades were relevant for reading and which were not. Future studies of microsaccades during natural reading should rely on more precise oculometry systems and systematically identify where in a sentence a microsaccade has landed.

## Conclusion

Our results indicate that increases in small saccadic intrusions with high spatio-temporal variability accompany slow-reading. These measures also accompany more deficient phonemic awareness, a key determinant of dyslexia. We thus conclude that intrusive fixational saccades seem to be an inherent part of slow reading and poor reading acquisition. Future studies should explicitly measure the perceptual span using gaze-contingent methods and establish a link between microsaccade proportion and spatiotemporal entropy on one side and the perceptual span’s characteristics on the other. Furthermore, we deem it fundamental to explore the electrophysiological correlates of intrusive fixational eye-movement generation during reading, and their relationship with the behaviorally measured perceptual span.

## Data Availability Statement

The raw data supporting the conclusions of this article will be made available by the authors, without undue reservation.

## Ethics Statement

The studies involving human participants were reviewed and approved by Newcastle University. The patients/participants provided their written informed consent to participate in this study.

## Author Contributions

SR conceived and designed the analysis, collected the data, performed the analysis, and wrote the manuscript. MS supervised the study, conceived and designed the analysis, and wrote the manuscript. Both authors contributed to the article and approved the submitted version.

## Conflict of Interest

The authors declare that the research was conducted in the absence of any commercial or financial relationships that could be construed as a potential conflict of interest.

## Publisher’s Note

All claims expressed in this article are solely those of the authors and do not necessarily represent those of their affiliated organizations, or those of the publisher, the editors and the reviewers. Any product that may be evaluated in this article, or claim that may be made by its manufacturer, is not guaranteed or endorsed by the publisher.

## Footnotes

1
www.okazolab.com
